# Simulating speech processing with cochlear implants: How does channel interaction affect learning in neural networks?

**DOI:** 10.1371/journal.pone.0212134

**Published:** 2019-02-27

**Authors:** Robert Grimm, Michèle Pettinato, Steven Gillis, Walter Daelemans

**Affiliations:** Computational Linguistics & Psycholinguistics Research Center, Department of Linguistics, University of Antwerp, Antwerp, Belgium; University of South Carolina, UNITED STATES

## Abstract

We introduce a novel machine learning approach for investigating speech processing with cochlear implants (CIs)—prostheses used to replace a damaged inner ear. Concretely, we use a simple perceptron and a deep convolutional network to classify speech spectrograms that are modified to approximate CI-delivered speech. Implant-delivered signals suffer from reduced spectral resolution, chiefly due to a small number of frequency channels and a phenomenon called *channel interaction*. The latter involves the spread of information from neighboring channels to similar populations of neurons and can be modeled by linearly combining adjacent channels. We find that early during training, this input modification degrades performance if the networks are first pre-trained on high-resolution speech—with a larger number of channels, and without added channel interaction. This suggests that the spectral degradation caused by channel interaction alters the signal to conflict with perceptual expectations acquired from high-resolution speech. We thus predict that a reduction of channel interaction will accelerate learning in CI users who are implanted after having adapted to high-resolution speech during normal hearing. (The code for replicating our experiments is available online: https://github.com/clips/SimulatingCochlearImplants).

## 1 Introduction

Cochlear implants (CIs) are neural prostheses that can partially restore hearing to individuals with sensorineural hearing loss. This type of hearing loss results from damage to sensory cells within the cochlea, an inner-ear organ that converts pressure waves into nerve impulses. CIs rely on an external microphone to capture sound signals from the environment and filter them into frequency bands whose amplitude envelopes modulate digital pulse sequences. These are then used to excite neurons through appropriately placed electrodes within the cochlea, where designated locations are responsible for processing specific frequency ranges.

The CI-delivered signal suffers from reduced spectral resolution, caused by (1) a limited number of electrodes; and (2) channel interaction [1, 2], which results from a group of neurons being stimulated by more than one electrode. Partly because of this, certain speech perception tasks present difficulties for CI users. For example, CIs make it harder to detect vocal emotion [3, 4], perceive tones [5], or to recognize speech in the presence of competing talkers [6, 7].

Recent work attempts to explain such task-specific differences in terms of the acoustic cues attended to by CI users and normally hearing (NH) subjects. For example, [8] found that the former have difficulties in telling apart speech which differs in fundamental frequency or vocal tract length of the speakers—cues which NH listeners rely on to distinguish pitch (fundamental frequency) and speaker height (vocal tract length). In a similar vein, [9] measured sensitivity to cues involved in the detection of phonemic contrasts. They report that CI users rely on coarse-grained cues related to the overall amplitude of the signal, rather than fine-grained differences between formants. NH subjects, in contrast, attend to both types of cues.

The performance differences between NH listeners and CI users, then, follow from differences in how the two populations process speech. And it stands to reason that differences in processing are, to some extent, a result of the input received by the learners. In other words, the fact that CI-delivered speech is characterized by a coarse spectral resolution, rendering more fine-grained features inaccessible, could conceivably push CI users to attend to coarse-grained acoustic cues; while NH individuals attend to coarse- *and* fine-grained features because the intact cochlea happens to transmits a greater level of spectral detail.

This, in turn, suggests that postlingually deaf CI users (PD-CI users), who receive CIs after a period of normal hearing, need to change the manner in which they process speech following implantation. This transition is likely to require neural re-wiring—which is presumably why the speech recognition performance of PD-CI users improves gradually, for well over a year, following implantation [10]. In the current study, we train neural networks under conditions that mimic those of PD-CI users, with initial exposure to high-resolution speech (corresponding to a period of normal hearing), followed by exposure to low-resolution speech (corresponding to the period after implantation). In doing so, we examine the effect of channel interaction on the transition from high- to low-resolution speech.

By *channel interaction*, we mean the (partial) summation of electrical fields generated by neighboring electrodes, leading to a distortion of amplitude envelopes for frequency channels assigned to specific electrodes, prior to neural activation [11]. This is an important cause of variability in speech recognition outcomes among CI users, with higher levels of channel interaction being associated with poor performance [12].

The mechanisms via which performance is affected clearly have to do with spectral resolution: In a task designed to measure the level of spectral detail utilized by subjects (spectral-ripple discrimination), higher levels of channel interaction were associated with poor outcomes [2]. Moreover, increasing the number of available electrodes past eight does not increase performance [1], suggesting that channel interaction makes adjacent electrodes less distinguishable. Thus, by decreasing spectral detail in the implant-delivered signal, channel interaction seems to inherently limit speech recognition performance.

Given this, one should naturally expect better performance if channel interaction was reduced. Here, we surmise that a reduction in channel interaction should *also* lead to faster learning in PD-CI users. Our basic argument is that post-implantation, PD-CI users may have to change the manner in which they process speech in order to accommodate the decreased spectral resolution in the implant-delivered signal. If channel interaction were eliminated, the signal would contain more detail, and PD-CI users would presumably have to make fewer adjustments to transition to speech processing with the implant.

To explore this, we train deep neural networks under conditions modeled on those faced by PD-CI users and CI users who are born deaf—and thus learn to process CI-delivered signals without first having adapted to an intact cochlea. In the following section, we specify our objective and method more closely. In section 3, we provide more details about the speech recognition tasks, data, and pre-processing steps. Finally, we present the results in sections 4—6, ending with a general discussion in section 7.

## 2 Research question and general method

Our research question can be phrased as follows: Does channel interaction slow learning during the period after implantation in PD-CI users, compared to congenitally deaf CI users (CD-CI users)?

In contrast to PD subjects, CD-CI users are born deaf—and, if implanted early enough, develop good speech recognition abilities [13]. Crucially, CD-CI users are only exposed to the degraded implant-delivered signal, while PD-CI users first learn to process speech delivered through the intact cochlea (while normally hearing) and then adapt to the CI after implantation. During the adaptation process, PD-CI users presumably integrate novel aspects of the CI-delivered signal—including the mode of neural stimulation (electrical current) and a reduction in spectral resolution. In this paper, we focus on spectral degradation caused by channel interaction, and we hypothesize that it slows adaptation to CIs in PD-CI users, with a comparatively smaller impact on learning in CD-CI users.

This effect could result from PD-CI users changing auditory processing strategies in order to transition from high-resolution input (delivered through the intact cochlea) to the spectrally degraded CI-delivered signal—for example, to emphasize coarse- rather than fine-grained spectral cues. Such a change in processing strategies is likely to require a certain amount of time. However, if the implant-delivered signal was less-coarse grained and thus more similar to high-resolution input, less time should be required, as that should reduce the amount of adaptation required on the part of PD-CI users. Thus, if we increase spectral resolution by reducing or removing channel interaction from the implant, we should expect faster adaptation to CIs.

Importantly, we expect this effect to be absent in CD-CI users: Since this population never learns to process speech delivered through the intact cochlea, they should be able to adapt to a degraded spectral resolution without having to modify existing processing strategies. Thus, while channel interaction should equally degrade maximum performance in PD- and CD-CI users *after* both populations have adapted to the implant, its impact on the learning process should be stronger in PD-CI users.

To gather evidence for the hypothesized effect, we train neural networks on (1) high-resolution (high-res) spectrograms (*X*_*h*_) with a large number of channels, intended as an approximation of what the intact cochlea delivers to the brain; (2) low-resolution (low-res) spectrograms, with a smaller number of channels suffering from channel interaction (*X*_*l*_), to approximate CI-delivered input; and (3) medium-resolution (med-res) spectrograms—essentially low-res spectrograms *without* channel interaction (*X*_*m*_), to approximate CI-delivered input if channel interaction was eliminated from the implants. We derive *X*_*h*_ by computing amplitude spectrograms with a large number of channels, *X*_*m*_ by constructing spectrograms with fewer channels, and *X*_*l*_ by linearly combining neighboring channels in *X*_*m*_.

We conduct experiments in a postlingually deaf (PD) condition, where we train first on *X*_*h*_, followed by further training on either *X*_*l*_ or *X*_*m*_; and a congenitally deaf (CD) condition, where we train only on *X*_*l*_ or *X*_*m*_. Networks trained on high-res speech should generalize poorly to *X*_*l*_ and perform better on *X*_*m*_. Given sufficient training on *X*_*l*_ and *X*_*m*_, CD and PD networks might eventually perform similarly on both input types. But at early epochs, before the PD networks have adapted to the decreased spectral resolution, we expect a larger performance difference in the PD condition.

This outcome would show that the spectral degradation introduced through channel interaction slows learning in deep neural networks as they adapt to low-res speech—*after they were pre-trained on high-res speech*. Assuming that deep learning is a reasonable model for pattern recognition in the brain, it would also suggest that decreased spectral resolution prevents PD-CI users from quickly adapting to CIs. Caution is surely required in making the connection to human processing; but since deep neural networks have proven useful for understanding brain-based sensory pattern recognition [14], we consider them as an exploratory tool for investigating learning dynamics with CIs.

## 3 Materials and methods

### Tasks and data

We train neural networks on gender and isolated word recognition, choosing the former because it presents difficulties for CI users [8, 15] and the latter because CI users can perform it with high accuracy [16]. If similar patterns appear across both tasks, despite the different performance patterns with CIs, we can be relatively confident in their robustness.

We frame gender recognition as binary classification of utterances into *male* or *female*, based on data from the Texas Instruments Massachusetts Institute of Technology (TIMIT) corpus [17]. TIMIT contains recordings of 630 speakers (70% male, 30% female) from 8 U.S. dialect regions. Each speaker read 10 different sentences, yielding 6,300 utterances spanning several seconds each (5.4 hours of speech).

The isolated word recognition task involves the classification of utterances into 30 word-classes, with data from the Google Speech Commands (GSC) corpus (https://research.googleblog.com/2017/08/launching-speech-commands-dataset.html, data were downloaded on 08/08/2018). Collected via crowd-sourcing, the GSC contains 65,000 one-second utterances of 30 short words (18 hours of speech). 20 core words were pronounced five times by most speakers, and an additional 10 words (treated as *unknown words*) were pronounced once. For each corpus, we use a randomly selected 20% of the data for validation, and another 20% for testing. All WAV files have a sampling rate of 16,000.

### Featurization

We train neural networks on input that is approximately similar to what the brain uses for speech recognition. Generally, the auditory system processes nerve impulses distributed across time and frequency—with the decomposition into distinct frequency components implemented mechanically by the inner ear (NH subjects), or digitally by the implant (CI users). Modern CIs work with 12—22 electrodes, and signals are decomposed into as many frequency components. The intact human cochlea, in contrast, contains thousands of sensory hair cells, where topographically coherent cell groups correspond to functional channels. The bandwidths of these cochlear channels have been investigated in various behavioral experiments, and the methods used continue to evolve [18]. In auditory models, 30 channels are often used to cover the frequency range relevant for speech [19, 20].

In this study, we approximate speech delivered through CIs and the intact cochlea via mel-scaled amplitude spectrograms (computed over windows of 50ms, strided by 10ms). Each spectrogram is a matrix with dimensionality *N* × *T*, where *N* is the number of channels and *T* is the number of frames, with *x*_*n*,*t*_ being the amplitude at time *t* and channel *n*. The channels, whose spacing and bandwidths conform to the perceptually motivated mel scale, cover the range between 200 and 7,000 Hz, similar to most CIs. A medium-resolution (med-res) condition with *N* = 16 channels approximates CI-delivered input in the hypothetical case that channel interaction was completely eliminated; and a high-resolution (high-res) condition with *N* = 32 channels approximates signals delivered through the intact cochlea.

We thus feed the networks with mel-scaled amplitude spectrograms, covering a frequency range similar to most CIs, with a number of channels similar to implants (med-res) or to the intact cochlea (high-res). In order to obtain low-resolution (low-res) input that approximates the signals transmitted through CIs, however, we still need to operationalize channel interaction—which can be approximated as a summation of potentials from individual electrodes [21]. Thus, given row *x*_*n*,*_ in a med-res spectrogram *x* with *N* frequency bands, we obtain a low-res spectrogram with added channel interaction by linearly combining rows:

$$\begin{array}{c} x_{n,*}=\{\begin{array}{cc} x_{n,*}+x_{n+1,*}, & \text{if}\;n=1\\ x_{n,*}+x_{n-1,*}, & \text{if}\;n=N\\ x_{n,*}+x_{n+1,*}+x_{n-1,*}, & \text{otherwise} \end{array} \end{array}$$

To ensure that data points from all three input conditions are represented as a 32 × *T* matrices, we duplicate each row in the low- and med-res spectrograms. Equally sized spectrograms are (1) necessary to train the same neural network on high- and either low- or med-res input and (2) roughly analogous to the contrast between the intact cochlea and CIs. With the latter, electrodes cover broad swaths of neurons; while in the intact cochlea, groups of sensory hair cells cover fewer neurons. Similarly, in the low- and med-res spectrograms, broad areas contain information from a single channel; and in the high-res spectrograms, smaller areas contain information from more narrow channels.

**Fig 1:**
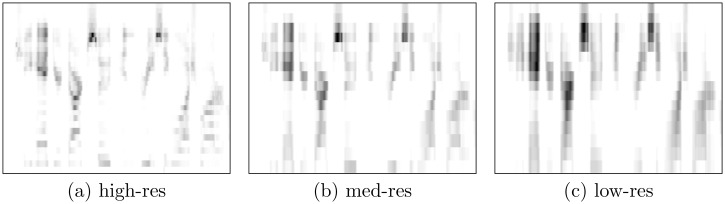
Example spectrograms (TIMIT corpus). Spectrograms based on a single utterance from the TIMIT corpus, in high-res (32 channels), med-res (16 channels), and low-res (16 linearly combined channels).

**Fig 2:**
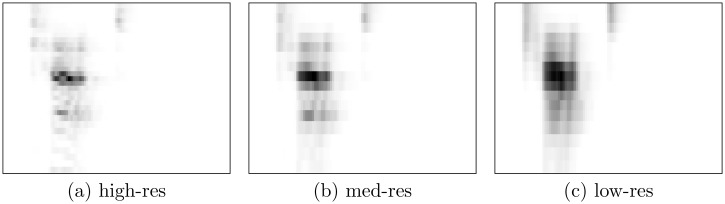
Example spectrograms (GSC corpus). Spectrograms based on a single word from the GSC corpus, in high-res (32 channels), med-res (16 channels), and low-res (16 linearly combined channels).

Note that our spectrograms approximate rather than simulate CIs. For example, the implants use amplitude envelopes to modulate digital pulse trains, whereas we use raw amplitude spectrograms. Due to the exploratory nature of this study, however, we abstract from additional complexity. See Figs 1 and 2 for example spectrograms.

### Network architectures

One of the simplest neural networks available is the perceptron (PER)—a flat architecture which directly classifies each input vector, without first transforming the input through one or more hidden layers. We implement the PER as a single softmax layer, with two neurons for gender recognition and thirty neurons for the word recognition task. Concretely, the probability that an input vector *x* is a member of class *i* (belonging to a stochastic variable *Y*, with two values for gender recognition and thirty values for word recognition) is be defined as:

$$\begin{array}{c} p\left(Y=i|x,\theta \right)=\frac{e^{W_{i}\cdot x+b_{i}}}{\sum_{j}e^{W_{j}\cdot x+b_{j}}} \end{array}$$ (1)

where the set of trainable parameters *θ* contains the weight matrix *W* as well as the bias *b*, and each input *x* is a spectrogram, converted from the original matrix into a vector format. This is done by concatenating the rows (= frequency bands) in a given spectrogram, with dimensionality *N* × *T*, to create an input vector with length *NT*. Due to its simplicity, the PER is an appealing model choice, but it also comes with disadvantages: (a) It is a flat architecture, limiting performance; and (b) each column in the weight matrix *W* and bias *b* corresponds to exactly one value in *x*, so that the patterns detected by the model are bound to particular input coordinates.

To obtain better performance, and to model the invariance of auditory processing to spectro-temporal details [22], we also report results with a deep convolutional neural network (CNN), trained to ingest spectrograms in matrix format. The first three layers contain 2D convolutions—which facilitate the detection of local patterns at different positions [23] by repeatedly applying sets of weights (filters) to *n* × *m* (filter size) sub-regions within the input, strided by *u* × *v* (stride). The convolutional layers are then followed by a fully connected layer, whose output is fed into a perceptron as in (1). For speedier convergence, we apply the Batch Normalization method [24] after each hidden layer, before the activation function is applied.

Since we compare performance on different featurizations of the same data (high-res, med-res, low-res), a selection of hyperparameters meant to optimize performance on any of the three featurizations could confound the results. To address this, we chose hyperparameters so that the validation error improves steadily, without noticeable fluctuations—but we do not tweak them for maximum performance. Since the CNN has a fairly large number of tunable hyperparameters, it is still possible that we accidentally picked settings which favor a specific featurization. For the PER, however, the only hyperparameters are the learning rate (0.01) and mini batch size (32), dramatically reducing this risk. Apart from the number of hidden layers and the application of batch normalization, hyperparameters for the CNN include the activation function (rectified linear function), regularization (0.1 dropout at each convolutional layer, 0.5 at the fully connected layer), number of filters in each convolutional layer (5), filter size (5 × 5), stride (2 × 2), number of hidden units in the connected layer (100), learning rate (0.1), and mini batch size (32).

For the CNN, we decided to keep the number of filters, filter size, and stride constant across successive layers in order to reduce the amount of fine-tuning required to get different architectures to work. This may raise the concern that our results are an artifact of the particular hyperparameters we have chosen. As mentioned, we use the PER to control for this possibility: Since the PER’s only hyperparameters are mini batch size and learning rate, we can be confident that the results are not confounded by our choices for number of filters, filter size, and stride if a similar pattern emerges with both the PER and the CNN.

The models are trained via mini batch gradient descent, on an Nvidia Titan X GPU, by minimizing the categorical cross-entropy of predicted and true class probabilities. The learning rate is adjusted via Adadelta [25], and training is terminated once the validation error has ceased to decrease for 10 epochs (early stopping with a patience of 10).

### Statistical testing

The key statistic of the current study is the difference in performance between neural networks trained on different featurizations of the same corpus. For example, given the TIMIT corpus, let *X*_*h*_ be a high-res featurization, and let *X*_*l*_ be a low-res featurization. We might train a network *A* on *X*_*h*_ and a network *B* on *X*_*l*_, obtaining accuracy scores *t*_*A*_, *t*_*B*_ by evaluating *A* and *B* on held-out portions of *X*_*h*_ and *X*_*l*_, respectively. The question of interest then is whether we can reject the null hypothesis that *t*_*A*_ = *t*_*B*_.

We can answer this via approximate randomization testing (ART), a simple approach that does not rely on assumptions about the data and is thus well-suited for application in machine learning [26, 27]. ART starts from the labels $C_{A}=\left\{c_{A}^{1},...,c_{A}^{n}\right\}$ and $C_{B}=\left\{c_{B}^{1},...,c_{B}^{n}\right\}$ assigned by the two networks to each data point in the held-out data. Each pair of labels $c_{A}^{i},c_{B}^{i}$ is then switched with probability $\frac{1}{2}$, and the difference in performance *d*′ is re-calculated. The procedure is repeated *R* times, with *r* being the number of times that *d*′ ≥ *d*. For large *R*, $p=\frac{r+1}{R+1}$ approximates the significance level. We set *R* = 10^5^.

Given *N* comparisons, we reject the null hypothesis if $p\leq \frac{0.05}{N}$. That is, we apply the Bonferroni method to correct for multiple comparisons, since these increase the chance of incorrectly rejecting the null hypothesis. In the analyses below, we conduct 18 model comparisons per corpus, obtaining a rectified significance threshold of $p\leq \frac{0.05}{18}$.

## 4 Analysis I: Preliminary comparisons

In this first analysis, we compare (1) the PER to the CNN and (2) performance on high-res spectrograms (32 channels) to performance on low-res spectrograms (16 channels with channel interaction). This serves as a sanity check: Due to the larger number of parameters and the location-independent, more generalizable features detected by the CNN, we expect worse performance with the PER; and due to the diminished level of spectral detail in the low-res featurization, we expect better performance on high-res input. Given our two network architectures (PER, CNN), the two featurizations (high-res, low-res), and the two tasks (gender, word recognition), we report results for 2 × 2 × 2 = 8 models.

### 4.1 Results and discussion

**Fig 3:**
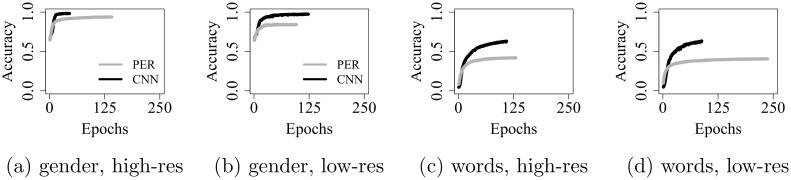
Validation accuracy over epochs, on high- and low-res spectrograms.

Fig 3 shows validation accuracy over training epochs. It is immediately apparent that the CNN outperforms the PER. Indeed, test accuracy achieved with the former is significantly higher: by ca. 10 – 15% for gender recognition, and by about 25% for word recognition (*p* ≤ 0.001). The larger performance gap on word recognition is likely due to a higher degree of spectro-temporal variability in the data used for this task, so that the CNN gains a comparatively stronger advantage.

Turning to the distinction between low- and high-res input (Table 1), we find that high-res spectrograms lead to better performance in three out of four model comparisons—as is expected, given the spectrally impoverished nature of the low-res spectrograms. The only non-significant difference emerges with the CNN, trained on word recognition (Table 1, last column). Here, we obtain a small negative difference, indicating that our particular instantiation of the CNN performs slightly better on low- than on high-res spectrograms. Given that this difference is not statistically significant, it is most likely due to chance—for example, it could result from chance differences in the randomly initialized network weights. The positive difference obtained with the PER (second to last column), in contrast, does reach significance—indicating that this less powerful architecture performs more poorly if the amount of spectral detail is reduced.

**Table 1:** Test accuracy, for networks trained on high- or low-res speech.

Task	Model	High-Res	Low-Res	Diff
gender	PER	93.1	84.3	**8.8** ***
	CNN	98.9	96.9	**2.0** **
words	PER	43.1	41.1	**2.0** ***
	CNN	63.1	63.5	-0.4

Diff = high-res accuracy minus low-res accuracy. The stars denote Bonferroni-corrected significance thresholds (*: *p* ≤ 0.05; **: *p* ≤ 0.01; ***: *p* ≤ 0.001).

With gender recognition, we find that both networks perform significantly better on high-res input, and that the differences are stronger than on the word recognition task. This indicates that loss of spectral detail is comparatively more detrimental for gender and less so for word recognition. These results make sense in light of performance patterns with CI users, who can solve isolated word recognition with high accuracy [16] but struggle with gender recognition [8]—suggesting that the former is more easily solvable with the spectrally impoverished CI-delivered signal.

## 5 Analysis II: Effect of channel interaction

We next compare the performance of networks trained only on med-res spectrograms (16 channels *without* channel interaction) or low-res spectrograms (16 channels *with* channel interaction). These training regimes are idealized analogs of the conditions faced by CD-CI users—equipped with hypothesized CIs that completely eliminate channel interaction (med-res spectrograms); or with CIs suffering from channel interaction, similar to the implants currently in use (low-res spectrograms).

Given that channel interaction in CIs is associated with poor speech recognition performance [2], we should likewise expect our operationalization of channel interaction to limit speech recognition performance in neural networks.

### 5.1 Results and discussion

**Fig 4:**
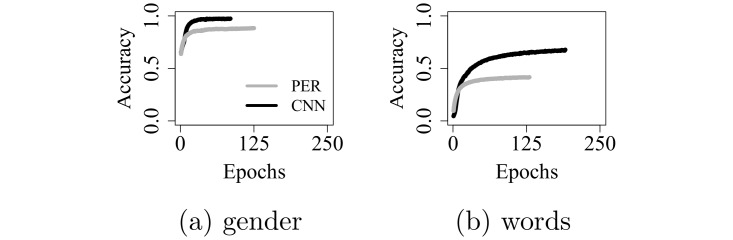
Validation accuracy over epochs, on med-res spectrograms.

Fig 4 shows validation accuracy over epochs, for networks trained on med-res data. Although the learning trajectories appear broadly similar to those obtained on low-res speech (Fig 3, subplots *b* an *d*), final test accuracy is significantly higher in the med-res condition, in three out of the four model comparisons: by 4.2% (PER, *p* ≤ 0.001) on gender recognition, as well as by 1.5% (PER, *p* ≤ 0.001) and 0.9% (CNN, *p* ≤ 0.05) on word recognition. For the CNN, no significant difference emerged when trained on gender recognition.

By reducing spectral detail, then, our operationalization of channel interaction limits accuracy. The observed performance degradations are generally weaker than the ones from the previous analysis, where we compared high- to low-res spectrograms. This is expected, since the difference (in spectral resolution) between the med- and low-res data is less strong than between high- and low-res input.

## 6 Analysis III: Effect of channel interaction with pre-training

In the foregoing analyses, we started training with randomly initialized parameters (weights and biases). Now, we use parameters that are pre-trained on high-res input (see Fig 3, subplots *a* and *c*), and we ask how well they generalize to either (1) low-res or (2) med-res spectrograms. (1) mimics the learning conditions of PD-CI users, who transition to the CI-delivered signal after having adapted to high-res speech during a period of normal hearing; and (2) mimics a hypothetical case where PD-CI users, having adapted to high-res input, transition to CIs that do not suffer from channel interaction.

As before, the networks come in two variants, applied to two different tasks. They are then trained on low-res or med-res spectrograms, yielding eight pre-trained models.

### 6.1 Results and discussion

**Fig 5:**
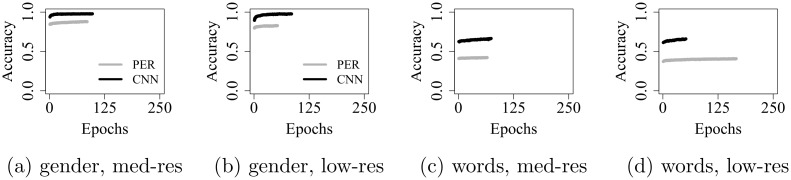
Validation accuracy over epochs, for pre-trained networks.

Fig 5 shows validation accuracy, on low- and med-res data, for models whose weights were pre-trained on high-res spectrograms. Notice how initial validation accuracy is already very high, compared to networks that were not pre-trained.

Without pre-training, the weights are randomly initialized, and accuracy is at chance level before training commences. (See Table 2, left side, bottom. Note that as in Table 1, some of the non-significant differences take small negative values. Since these small negative differences fail to reach statistical significance, they are most likely due to chance. They could, for example, result from chance differences in the randomly initialized network weights.) The parameters acquired from high-res input, however, generalize to both the med- and low-res data, affording high initial performance (left side, top). Table 2 shows, moreover, that the pre-trained parameters generalize better to the med-res data, with significantly lower initial accuracy on low-res input—both before training commences, as well as early during learning (after the first epoch). Once training has finished, the pre-trained networks still generally perform worse on low-res input, but the differences are not as strong as early during training.

**Table 2:** Test accuracy, for pre-trained (top) and randomly initialized (bottom) networks.

Init.	Task	Model	med-r.	low-r.	Diff	med-r.	low-r.	Diff	med-r.	low-r.	Diff
pre-tr.	gender	PER	83.0	75.3	**7.7** ***	85.1	79.4	**5.7** ***	87.3	83.1	**4.2** ***
		CNN	74.6	73.1	1.5	93.5	90.0	**3.5** ***	97.6	97.3	0.3
	words	PER	41.0	36.5	**4.5** ***	41.5	38.0	**3.5** ***	42.6	41.1	**1.5** ***
		CNN	62.7	58.4	**4.3** ***	62.6	62.6	**1.0** ***	66.7	65.8	**0.9** *
rand.	gender	PER	50.3	51.1	-0.8	63.4	63.0	0.4	87.1	84.3	**2.8** **
		CNN	59.2	59.2	0.0	65.8	63.4	2.4	96.2	96.9	-0.7
	words	PER	2.9	2.6	0.3	9.7	9.7	0.0	42.4	41.1	**1.3** *
		CNN	3.3	3.5	-0.2	5.1	5.0	0.1	67.0	63.5	**3.5** ***

Left: accuracy before training on med- or low-res spectrograms. Middle: accuracy after the first epoch. Top: accuracy after the final epoch. Diff = accuracy on med-res spectrograms minus accuracy on low-res spectrograms. The stars denote Bonferroni-corrected significance thresholds (*: *p* ≤ 0.05; **: *p* ≤ 0.01; ***: *p* ≤ 0.001).

The opposite pattern emerges for the randomly initialized models: Initially, these models make random guesses, regardless of input conditions. But after training has run its course, significant differences emerge. For the randomly initialized models, in other words, differences between med- and low-res input emerge and *increase* over time, whereas they are present from the beginning and *decrease* over time if the parameters are pre-trained on high-res spectrograms.

This can be explained by differences in spectral resolution between the input conditions. The pre-trained weights, being optimized for processing high-res spectrograms, generalize better to med- than to low-res spectrograms because the resolution of the former is closer to high-res input. After training, the strong initial differences between the med- and low-res conditions decrease, suggesting that the pre-trained networks compensate, to some extent, for the reduced resolution. But early during training, when they process low-res speech as if it was high-res speech, the stronger spectral degradation severely degrades performance.

The degraded spectral resolution in the low-res data is a direct result of channel interaction (which is absent in the med-res spectrograms). Thus, not only does our operationalization of channel interaction lead to reduced accuracy *after* training, but it also leads to slower initial performance gains for the pre-trained networks. These results imply that channel interaction in CIs should not only limit speech recognition performance after CI users have fully adapted to the implants, but that it should *also* slow learning in PD-CI users during the transition period after implantation.

## 7 Conclusions

In an effort to investigate the impact of channel interaction on learning in CI users, we trained neural networks on two types of spectrograms, intended to approximate CIs with and without channel interaction. We generally obtained poorer performance on the former—in spite of training the networks for as long as is necessary for performance to plateau. This suggests that channel interaction leads to the irrecoverable loss of crucial spectral detail, corroborating previous findings that CI users perform worse in the presence of channel interaction due to a decrease in spectral resolution [1, 2].

Apart from a performance degradation after training, we also found a negative impact of channel interaction on the early performance of networks pre-trained on spectrograms intended to approximate the speech delivered through the intact cochlea (high-res input). We observed that the effect was absent in models that were not pre-trained; and that the pre-trained networks recovered with additional training, until they performed similarly to their randomly initialized counterparts. This second effect, then, arises only if the models have adapted to high-res input, and it is reversible over time.

The implication for CIs is that spectral degradation caused by channel interaction should slow learning in PD- but not in CD-CI users. Prior to implantation, only the former adapt to normal hearing; and this might force them to unlearn certain processing strategies that may be applicable to normal hearing—but that do not generalize to CIs due to the impoverished nature of the implant-delivered signal. For example, PD-CI users might need to re-learn which acoustic features they attend to, with more emphasis on coarse- rather than fine-grained features after implantation. If spectral resolution is increased, there should be less need for such adaptation. Consequently, we predict that techniques for the reduction of channel interaction [28] will accelerate speech recognition improvement in PD-CI users.

More generally, our study demonstrates how machine learning can be used to shed light on questions in the field of CI research. Our approach allows us to quickly evaluate the impact of input modifications on auditory pattern recognition, without the difficulties involved in conducting behavioral studies (e.g. time constraints, ethical considerations). In similar machine learning experiments, other input properties could be examined. For example, one could model the electrical pulse trains generated by CIs and investigate how the pulsatile nature of the signal affects processing.
